# Olfactory dysfunction in children is associated with atopic dermatitis and allergic rhinitis, but not bronchial asthma 

**DOI:** 10.5414/ALX02644E

**Published:** 2026-09-10

**Authors:** Elisabeth C. Lohrer, Janine Gellrich, M. Antonia Hinkelmann, Rene F. Drosdek, Christian Vogelberg, Valentin A. Schriever

**Affiliations:** 1 Department of Neuropediatrics, Department of Pediatrics,; 2 Department of Pediatrics, Faculty of Medicine and University Hospital Carl Gustav Carus, Technische Universität Dresden, Dresden, and; 3 Goethe University Frankfurt, Department of Pediatrics, Division of Pediatric Neurology, Neurometabolics and Prevention, Frankfurt (Main), Germany; *These authors contributed equally.

**Keywords:** olfaction, atopic dermatitis, bronchial asthma, allergic rhinitis, olfactory dysfunction, hyposmia, children

## Abstract

Aims: To investigate associations between olfactory dysfunction and atopic dermatitis (AD), allergic rhinitis (AR), and bronchial asthma (BA) in children. Materials and methods: In a prospective study, 250 children (6 – 17 years) with AD, AR, BA, combined atopy, or healthy controls (n = 50 each) were assessed using olfactory tests (“Sniffin’ Sticks”, “U-Sniff”), questionnaires, prick tests, and spirometry. Results: Atopic patients showed poorer odor identification than controls. Children with AD had impaired odor identification. AR patients displayed more hyposmia in threshold testing, but mean scores did not differ significantly. BA patients showed no significant impairment, though lung function correlated positively with olfactory performance. Conclusion: Pediatric AD and AR are associated with specific olfactory dysfunctions, whereas BA is not. Awareness of olfactory deficits in these groups is warranted; further studies should assess neurodevelopmental aspects in AD.

## Introduction 

Atopic diseases, including atopic dermatitis (AD), bronchial asthma (BA), and allergic rhinitis (AR), represent a significant global health burden in pediatric populations. These conditions are highly prevalent, with AD having the highest lifetime prevalence (12.8%), followed by BA (6 – 12.7%) and AR (11%) [1, 2, 3]. Typically triggered by aeroallergens or contact allergens, these diseases lead to characteristic symptoms such as shortness of breath in BA, rhinitis in AR, or eczema in AD [1, 2, 3]. The “atopic march” describes their common age-distributed onset, often beginning with AD in infancy and progressing to AR and/or BA in later childhood or adolescence. However, these diseases can manifest individually and to different timepoints or in combination [1, 4]. 

While several studies have linked AR to olfactory dysfunction in both adults and children [5, 6, 7, 8, 9, 10], evidence for such a trend in children and adults with BA, particularly without comorbidities, is limited [11, 12, 13]. Despite the high prevalence of atopic diseases and the known impact of olfactory dysfunction on quality of life [14, 15], there is a notable lack of comprehensive investigations into the frequency of olfactory disorders in children and adolescents with various combinations of atopic diseases. Olfactory function significantly impacts aspects such as finding a partner, health, and safety (e.g., smelling smoke or gas) [16, 17]. 

Therefore, this study aims to investigate the relationship between olfactory disorders and BA, AR, and AD. For this purpose, the following questions were formulated:

- Does the olfactory function of children and adolescents with atopy (BA, AR, and AD) differ from a healthy control group?
- Do olfactory disorders occur more frequently in children and adolescents with the complete picture of atopy than in children and adolescents suffering from one atopic disease only?
- Does the severity of AR (ARIA criteria), BA (Spirometry, C-ACT) and AD (SCORAD) influence olfactory function?

## Material and methods 

This prospective controlled study was approved by the Ethics Committee of the Medical Faculty at TU Dresden (BO-EK-197052020) and was conducted in accordance with the Declaration of Helsinki. Informed consent was obtained from legal guardians and verbal assent from participants after they were given thorough verbal and written information about the study. Pseudonymization safeguarded participants’ data privacy. 

A total of 250 participants aged 6 – 17 years were included, comprising five groups of 50 participants each: AD, AR, BA, combined atopy (AD/AR/BA), and a healthy control group. The sample size was determined based on a prior study to assess olfactory ability via ANOVA with a mean effect size of 0.3, an α-error of 0.05, and a power of 0.9. 

Participants underwent detailed medical history questioning using standardized questionnaires, as well as psychophysical olfactory testing, skin prick tests and spirometry. 

### Questionnaires 

Asthma control was assessed using the Childhood Asthma Control Test (C-ACT) [18]. While the C-ACT is validated for children aged 4 – 11 years, we opted to utilize this 7-item questionnaire across our entire study cohort (up to 17 years of age). This approach was chosen to ensure internal consistency and to allow for direct, quantitative comparisons across the full age range of our participants, thereby avoiding instrument-based confounding that would have arisen from using different assessment tools for younger and older patients. 

AR was categorized according to symptom duration and severity using the AR and its Impact on Asthma (ARIA) criteria [19]. Symptom severity was also quantified using the Total Symptom Score 4 (TSS4) (0 – 12 points; higher scores indicating more severe symptoms) [20, 21]. 

AD severity was quantified using the SCORAD (Scoring of Atopic Dermatitis) index, which evaluates extent, intensity and subjective symptoms (0 – 103 points) [22]. 

### Olfactory testing 

Olfactory function was assessed using the validated “Sniffin’ Sticks” threshold subtest (phenylethyl alcohol, 16-point scale) [23] and the “U-Sniff” odor identification test for children (12 odors, 0 – 12 points) [24]. The odorants were presented in felt-tip pens (“Sniffin’ Sticks”, Burkhardt GmbH, Holm, Germany). 

For the olfactory threshold test, a rose-like odor – phenylethyl alcohol – was used. The test was performed in a staircase fashion, starting with the highest dilution of phenylethyl alcohol. The test consisted of 16 triplets of Sniffin’ Sticks. One felt-tip pen of each triplet contained the dilution of phenylethyl alcohol, while the other 2 pens were odorless. Subjects were blindfolded to prevent visual identification of the odorant pen. Using a 3-alternative forced-choice paradigm, the children had to identify the odorant pen from the triplet. After 2 correct or 1 incorrect identification, the next higher or lower threshold staircase was elicited. The staircase procedure was repeated until 7 reversal points were reached. The average of the last 4 reversal points provided the individual olfactory threshold. 16 points was the maximum score that could be obtained in the olfactory threshold test [25]. Higher scores indicate better olfactory sensitivity (i.e., lower odorant concentration required for detection), and lower scores indicate reduced sensitivity (i.e., higher odorant concentration required for detection). 

In addition, a pediatric suprathreshold test, the “U-Sniff” odor identification test, was used [24]. Each of the 12 odorants was administered separately. The task was to identify the odors using 4 descriptors presented in pictures and text. If a participant was unable to read, the experimenter read aloud the cards and showed the labeled pictures. The interval between odor presentations was ~ 20 seconds. The number of odors correctly identified was summed to produce an odor identification score. Participants’ scores could range from 0 to 12 [24]. 

### Prick test 

Type I sensitization was determined via skin prick tests on the volar side of the forearm [26]. 15 standardized allergen solutions, a histamine positive control and a saline negative control were applied. A mean wheal diameter of ≥ 3 mm indicated a positive reaction after 15 minutes. Participants refrained from taking antihistamines and applying topical glucocorticoids for 7 days before the test. Sterile prick test lancets and allergen solutions (numbers: 006, 108, 115, 129, 154, 158, 169, 309, 400, 401, 412, 708, 725, 901, 902) from Allergopharma GmbH & Co. KG (Reinbek, Germany) were used. 

### Spirometry 

Lung function was measured using a Flowscreen (Erich Jaeger GmbH & Co. KG, Würzburg, Germany) [27]. This assessment evaluated static and dynamic lung volumes and flow rates, including vital capacity (VC), forced expiratory volume in 1 second (FEV_1_), and maximal expiratory flows (MEF_25_ and MEF_50_). Spirometry was performed using a Flowscreen device from Erich Jaeger GmbH & Co. KG. MicroGard IIB bacteria/virus filters from Vyaire Medical GmbH (Hoechberg, Germany) were used as mouthpieces, and disposable nose clips from Medisize Deutschland GmbH (Siegburg, Germany) were used as nose clips. 

### Data analysis 

Statistical analysis was conducted using SPSS version 28.0.0.0 (IBM Corp., 2021). Descriptive statistics (mean ± SD and SEM) were calculated for all test parameters. Normality was assessed using the Kolmogorov–Smirnov test, and homogeneity of variances was evaluated using Levene’s test. χ^2^-squared tests and analyses of covariance (ANCOVA), with age as a covariate, were performed. Where applicable, due to non-normal distributions, data were bootstrapped (1,000 samples). Pearson’s correlation analysis was used to explore the relationships between the variables. Significance was set at p ≤ 0.05. 

## Results 

### Study population 

A total of 250 children (6 – 17 years) were included (n = 50 per group: AD, AR, BA, combined atopy, healthy controls). The average age was 11.08 ± 3.41 years. A Kruskal-Wallis test indicated significant age differences between the groups (H(4) = 20.02, p < 0.001), with the AD group being younger than the others. Sex distribution was balanced (52% boys, 48% girls) with no significant differences between groups (χ^2^(4) = 3.21, p = 0.52) (Table 1). 

### Olfactory function across study groups 

Patients with atopic diseases showed a mean olfactory threshold of 9.4 ± 3.3 and an identification score of 10.2 ± 1.7, compared to 9.3 ± 3.5 and 11.0 ± 1.4 in healthy controls. An age-corrected analysis confirmed a significant impairment in odor identification for atopic patients compared to healthy controls (F(1,247) = 8.772, p = 0.003). 

When analyzing the five study groups separately, no significant differences were observed regarding olfactory thresholds (Table 2) (Figure 1). All groups demonstrated comparable sensitivity to odorants. Conversely, odor identification performance differed significantly between groups (Table 2). Post-hoc analysis identified that children with AD had significantly lower identification scores compared to healthy controls (Figure 2). No other significant differences were found between the remaining atopic subgroups and the control group. 

### Prevalence of olfactory dysfunction 

Overall, 24.4% (n = 61) of all children were classified as hyposmic regarding olfactory thresholds, and 10.8% (n = 27) regarding odor identification. While the control group showed the lowest rate of threshold impairment, the AR group exhibited a significantly higher prevalence of reduced olfactory threshold (p = 0.02, Fisher’s exact test). All other comparisons of hyposmic rates did not reach statistical significance. 

### Subgroup analysis atopic dermatitis 

Within the AD cohort (n = 50), the 6 children who exhibited odor identification deficits had a mean age of 11.67 ± 3.5 years and a mean SCORAD of 23.35 ± 15.2. In comparison, the remaining 44 children in the AD cohort had a mean age of 8.84 ± 3.2 years and a mean SCORAD of 27.90 ± 15.4. Statistical analysis revealed no significant differences in age (p = 0.8) or disease severity (p = 0.8). This indicates that olfactory impairment in our pediatric AD cohort is not primarily driven by age or disease severity (SCORAD), suggesting that other factors, such as individual neuro-sensory sensitivity or specific inflammatory profiles, may contribute to these findings. 

### Correlation of olfactory function and clinical parameters 

We performed Pearson’s correlation analysis to investigate associations between olfactory function (threshold/identification) and clinical disease severity scores (spirometry, C-ACT, ARIA, TSS4, SCORAD) as well as allergen sensitization. 

Results are summarized in Table 3. We observed significant positive correlations between lung function parameters – specifically VC, FEV_1_, MEF_25_, and MEF_50_ – and both olfactory threshold and identification performance (all p < 0.05). In contrast, no significant correlations were found between olfactory function and disease-specific activity scores (C-ACT, ARIA, TSS4, SCORAD) or individual allergen sensitization patterns (p > 0.05). These findings suggest that general lung function parameters may reflect systemic physiological aspects related to olfactory sensitivity, whereas acute disease-specific severity scores (like SCORAD or C-ACT) do not show a direct linear association with olfactory performance in our cohort. 

## Discussion 

The present study investigated whether children with atopic diseases are more commonly affected by olfactory dysfunction than healthy controls and if this is linked to disease severity. Overall, atopic patients showed no significant age-corrected difference in olfactory threshold compared to controls, a finding consistent when individual atopic groups were compared to healthy controls. 

Regarding AR, numerous studies suggest olfactory dysfunction in this patient group [8, 28]. While our analysis of mean olfactory scores did not show a significant difference, the absolute numbers revealed a significantly higher prevalence of a reduced olfactory threshold hyposmia in children with AR compared to healthy controls using Fisher´s exact test. Two primary mechanisms might lead to a reduced olfactory function. One is the mechanical blocking, i.e., nasal obstruction by mucous edema and/or secretion. The second is an affection of the olfactory bulb because of inflammation [28, 29, 30]. The severity and duration of the symptoms seem to correlate with olfactory dysfunction [28]. Assessments of AR patients during symptomatic phases showed that standard-of-care medications (antihistamines, nasal steroids) did not significantly influence olfactory scores, supporting the validity of our findings across the active disease spectrum. However, there seems to be no effect of local therapy on olfactory function [28, 31]. 

One potential, albeit speculative, explanation for the absence of correlation between AR severity scores and olfactory dysfunction in this study compared to other studies could be the duration of the disease. Given that we did not systematically record disease duration in our cohort, we hypothesize that a shorter overall disease history in our patients may have influenced the severity of the observed olfactory deficits. Future studies with longitudinal data on disease chronicity are needed to confirm this. 

This may also contribute to the absence of a significant difference in mean olfactory scores between AR patients and healthy controls, despite the higher prevalence of hyposmia observed. As AR prevalence increases with age, more patients are expected to present with ongoing symptoms [9, 10]. 

Similarly, regarding BA, there was no difference between groups. Only one study in adults shows a link between olfactory dysfunction and BA, but also identifies AR as a possible co-factor of olfactory disturbance [13]. Other studies also indicate that the presence of AR is the primary cause of olfactory disturbance in patients with BA. [12, 32]. One possible explanatory approach is that the inflammation in BA is localized mainly in the bronchial parts of the lung [33], potentially leading to less affection of the olfactory bulb. 

Nevertheless, we observed positive correlations between lung function parameters (VC, FEV_1_, MEF_25_, MEF_50_) and both odor identification and olfactory threshold. These parameters, indicative of lung function, suggest that better lung function correlates with better olfactory identification and also with better olfactory thresholds (i.e., higher threshold scores). This aligns with the expectation that overall health status might impact sensory functions. This suggests a subclinical effect of bronchial inflammation on olfactory function, even if not detectable as a significant group difference in mean olfactory testing scores. 

Our results showed a decreased odor identification ability in patients with AD. Previously, various studies have suggested potential links between severe pediatric eczema, chronic sleep fragmentation, and neurodevelopmental parameters [34]. However, in our study, the number of AD children exhibiting specific odor identification deficits was small (6 out of 50). Therefore, we cannot establish a definitive neurocognitive link based on these data. Instead, a potential connection between severe AD, chronic pruritus, sleep disturbance, and cognitive olfactory tasks as discussed in related clinical frameworks [1] remains strictly hypothetical at this stage. Our findings should be regarded as an exploratory observation that warrants further investigation in larger, dedicated prospective cohorts to determine whether these olfactory deficits are transient or reflect a long-term neurodevelopmental impact. While the exact mechanisms remain unclear, we acknowledge that the heterogeneity of the AD cohort, including varying degrees of disease severity, may influence sensory processing. Consequently, our data do not suggest a generalized neurocognitive deficit, but rather highlight the need for more granular, longitudinal studies that combine olfactory testing with objective sleep and itch assessments. 

A potential limitation of our study is the use of the C-ACT questionnaire for participants aged 12 – 17 years, although it is strictly validated for children aged 4 – 11 years. We acknowledge this as a minor methodological deviation. However, we prioritized this standardized approach to maintain comparability across our diverse age groups. We believe this choice is justified by the need for a uniform assessment tool to provide consistent data across the entire pediatric and adolescent spectrum, and our results are unlikely to have been significantly biased by this decision. 

In summary, this study demonstrates that children with AD are significantly more affected by impaired odor identification, and those with AR show a higher prevalence of reduced olfactory threshold. Further studies are also needed to detect, whether there is an influence on quality of life throughout olfactory dysfunction in patients with atopic diseases, a complication that requires increased awareness and knowledge, its detection and treatment options. Nevertheless, further studies are needed to understand the reasons why children with AD experience decreased odor identification, involving the testing of neurodevelopmental outcome parameters. 

## Data availability 

The data underlying this article cannot be shared publicly due to the data protection law of the country of origin. 

## Authors’ contributions 

Elisabeth C. Lohrer: Writing the manuscript, critical revision, and final approval of the submission. 

Janine Gellrich: Conceptualization of the research topic, writing the manuscript, critical revision, and final approval of the submission. 

M. Antonia Hinkelmann: Data collection for the study as a doctoral candidate and critical revision. 

Rene F. Drosdek: Data collection for the study as a doctoral candidate and critical revision. 

Christian Vogelberg: Conceptualization of the research topic, critical revision of the manuscript, and final approval of the submission. 

Valentin A. Schriever: Conceptualization of the research topic, critical revision of the manuscript, and final approval of the submission. 

All authors have read and approved the final version of the manuscript and confirm that they agree with the submission. 

## Funding 

JG was supported by a scholarship for the habilitation from the Medical Faculty Carl Gustav Carus in Dresden as well as a grant from Gesellschaft für Pädiatrische Allergologie und Umweltmedizin. 

## Conflict of interest 

None. 

**Table 1.** Sociodemographic characteristics of the study population. Data are presented as mean ± SD or absolute numbers (n).

	**AD**	**AR**	**BA**	**Combined**	**Control**
Age	9.2 ± 3.3	11.4 ± 3.2	11.5 ± 3.3	11.7 ± 3.3	11.7 ± 3.3
Sex distribution (male : female)	24 : 26	31 : 19	25 : 25	27 : 23	24 : 26

AD = atopic dermatitis; AR = allergic rhinitis; BA = bronchial asthma; Combined = combination of atopic diseases.

**Table 2.** Olfactory performance across study groups, data are age-adjusted estimated marginal means (EMM) ± standard error (SE). ANCOVA results for threshold: F(4,244) = 0.67, p = 0.612, ηp2 = 0.011. ANCOVA results for odor identification: F(4,244) = 2.56, p = 0.039, ηp2 = 0.040.

**Parameter**	**AD**	**AR**	**BA**	**Combined**	**Control**
Olfactory (Thr)	8.8 ± 0.5	9.9 ± 0.5	9.6 ± 0.5	9.4 ± 0.5	9.2 ± 0.5
Odor (Id)	10.0 ± 0.2	10.1 ± 0.2	10.3 ± 0.2	10.3 ± 0.2	10.9 ± 0.2
Hyposmic (Thr)	10	18	13	13	7
Hyposmic (Id)	6	4	3	8	6

AD = atopic dermatitis; AR = allergic rhinitis; BA = bronchial asthma; Combined = combination of atopic diseases; Thr = threshold; Id = identification.

**Figure 1.**
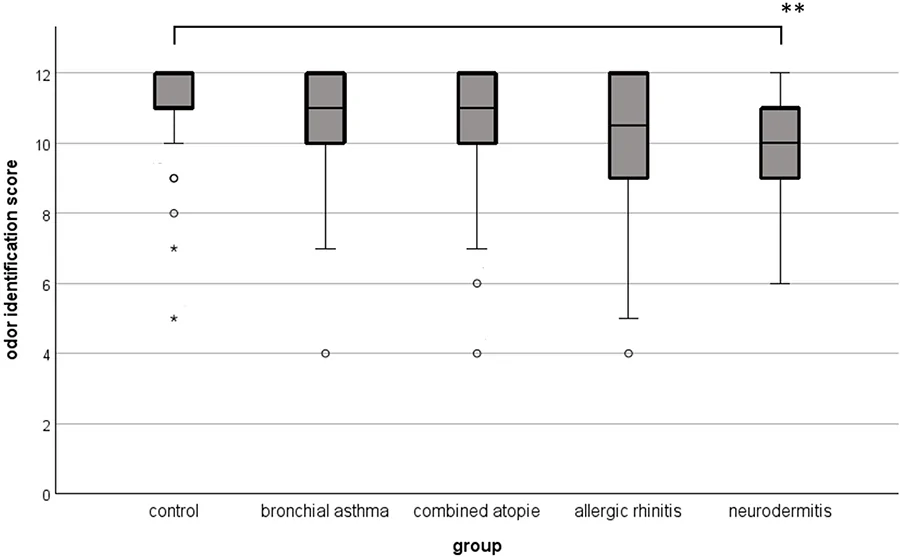
Odor identification scores across study groups. Boxplots represent the median and interquartile range of identification performance. Significant differences compared to the healthy control group are indicated by an asterisk (**p < 0.001, Bonferroni-corrected post-hoc test).

**Figure 2.**
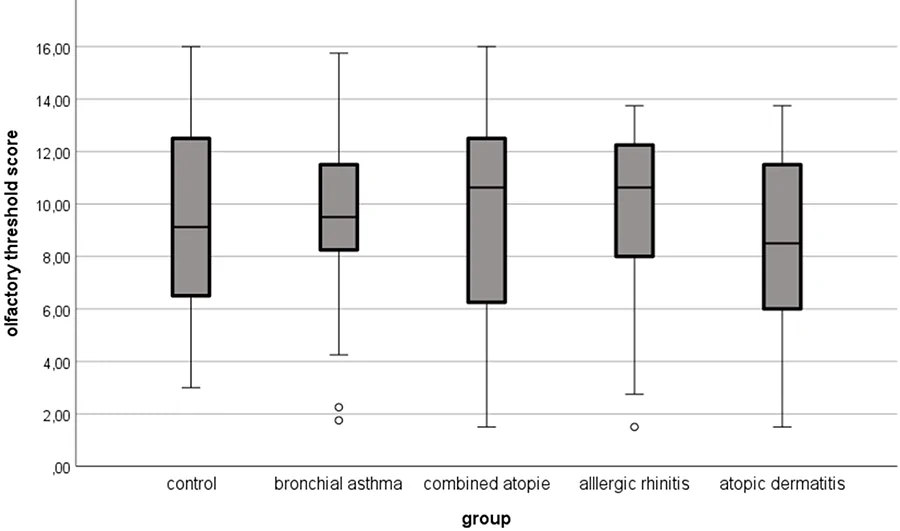
Olfactory threshold scores across study groups. Boxplots display the median and interquartile range of the age-adjusted threshold scores. No statistically significant differences were observed between the study groups (p > 0.05).

**Table 3.** Correlation analysis between clinical parameters and olfactory function. Pearson’s correlation coefficients (r) and corresponding p-values are shown.

**Parameter**	**Olfactory threshold (r)**	**p-value**	**Odor identifikation (r)**	**p-value**
Vital capacity	0.143	0.025	0.324	< 0.001
FEV_1_	0.144	0.026	0.320	< 0.001
MEF_25_	0.203	0.014	0.294	< 0.001
MEF_50_	0.248	0.002	0.305	< 0.001
SCORAD	–0.047	0.646	0.002	0.987
C-ACT (total)	–0.072	0.475	-0.105	0.297
ARIA score	0.005	0.954	-0.055	0.503
TSS4 score	–0.06	0.463	-0.129	0.115

Significant correlations are highlighted in bold.
